# Editorial: Methods in aquatic microbiology

**DOI:** 10.3389/fmicb.2023.1338297

**Published:** 2023-12-04

**Authors:** Tony Gutierrez, Frederic Coulon, Kai Ziervogel

**Affiliations:** ^1^School of Engineering and Physical Sciences, Institute of Mechanical Process and Energy Engineering (IMPEE), Heriot-Watt University, Edinburgh, United Kingdom; ^2^School of Water, Energy, and Environment, Cranfield University, Cranfield, United Kingdom; ^3^Institute for the Study of Earth, Oceans, and Space, University of New Hampshire, Durham, NC, United States

**Keywords:** methods, aquatic microbiology, sampling, sequencing, phylogenetics

This Research Topic highlights the latest experimental techniques and methods used to investigate fundamental questions in Aquatic Microbiology research, from aquatic microbe sampling and culturing, to sequencing, phylogenetics, and microbial material cycling. The Topic includes new or existing methods/technologies that have been significantly improved or adapted that help advance our understanding of the identification and characterization of microbial species and ecosystems in aquatic environments. We hope that this Research Topic will feed into existing research, stimulate new research and collaboration in the coming years.

It includes a total of 11 articles, with three articles describing new methods to sample and improve prokaryotic or eukaryotic community surveys in aquatic systems. To begin, the article by Ma et al. investigated if bias is introduced into eukaryotic plankton community assessments based on DNA-based community sequencing when using size-fractionation (i.e., different filter pore sizes) when sampling. The authors showed that this can indeed lead to significant differences in community composition, and that this had more influence on unique OTUs than shared OTUs, and on low-abundance OTUs. In the article by Ranchou-Peyruse et al. the authors developed an improved method for sampling of deep aquifers to eliminate contamination. Microbial sampling of deep aquifers is challenging because of the need to minimize, and ideally eliminate, contamination from surface microbial contaminants, as this is key to guaranteeing the best interpretations and understanding of the functioning of the deep biosphere. In this study, the adaptation, preparation, sterilization, and deployment of a commercial downhole sampler (PDSshort, Leutert, Germany) for studying the microbiology of deep aquifers, is presented. The authors showed that this improved sampler allowed the formation water and the autochthonous microbial community to be maintained at *in situ* pressure for laboratory analysis. To improve the automation and time-scale resolution for sampling and data collection to assess prokaryotic community structure during research cruises, the paper by Pernice and Gasol showed the successful coupling of an automated continuous water sampler (OC-300) that samples marine surface waters every 15 minutes and stains the microbial cell community, with a flow-cytometer (Accuri-C6) that then quantifies the community based on size. The method would be an excellent add-on to sequencing surveys as it provides absolute values for community abundances.

To drive the discovery of new biotechnology products from previously unculturable microbes, several methods such as modification of media composition, incubation conditions, single-cell isolation, and *in situ* incubation, have been employed to improve microbial recovery from environmental samples. Here, three papers present new methods to isolate and culture marine bacteria. To improve microbial recovery, the article by Pope et al. examined the effect of microencapsulation followed by *in situ* incubation on the abundance, viability, and diversity of bacteria recovered from marine sediment. The authors showed that encapsulation recovered greater bacterial diversity from the sediment than simple resuspension (41 vs. 31 OTUs, respectively), and overall this study provides another tool that microbiologists can use to access microbial dark matter for environmental, biotechnology bioprospecting. With respect to the latter, to exploit the microbes present in the environment for their beneficial resources, effective selection and isolation of microbes from environmental samples is essential. In the study by Duran et al. the authors used a gel-filled microwell array device, using resin for microbial culture, that was fabricated and validated for isolating and culturing marine bacteria in individual wells. The design essentially comprises a simple, effective and cheap device for the first-step screening of microorganisms from marine environmental samples. In the article by Jung et al. two advanced methods, based on a continuous-flow bioreactor (CF) and *in situ* cultivation (I-tip), were evaluated to isolate previously uncultivated marine sponge-associated bacteria. This approach was found to yield a greater number of novel bacteria compared to conventional direct plate cultivation. To understand the mechanisms operating in each cultivation method, and which could help further improve the methodology, the physiological properties of the isolates from each method were characterized. From this, it was found that some of the bacteria require a “growth initiation factor” that is present in the natural environment, and that it would be beneficial to carry out pre-enrichment cultivation targeting bacteria that are less competitive on conventional cultivation. This work provides a step forward in cultivation methods together with improved understanding of the key factors for cultivating previously uncultivated microbes which can be applied to build new cultivation strategies, and that can be integrated into discovery pipelines for isolating microbes that are sources for valuable secondary metabolites of biotechnological interest.

In some facets of aquatic microbiology, such as in aquaculture, the ability to detect low numbers of microbial cells in water or tissue samples is highly valuable but remains a significant challenge. This Research Topic presents two articles that describe new or improved methods that address this challenge. The article by Luo et al. presents a new method for the detection of *Prymnesium parvum* – a harmful algal bloom (HAB)-forming species that produces a collection of compounds known as prymnesins that can cause harmful effects to fish, shellfish, and molluscs, resulting in significant economic losses. The novel method described in this study utilizes isothermal amplification, known as recombinase polymerase amplification (RPA), in combination with lateral-flow dipstick (LFD). The authors showed the newly designed RPA-LFD method to be highly specific, demonstrating no cross-reaction with distinct control microalgae. It is 100 times more sensitive than the current PCR-based detection test for *P. parvum*, and the test result can be obtained in 20 min. The report by Geraci-Yee et al. describes the development of a detection assay for Quahog Parasite Unknown (QPX) disease in hard clams. This disease has caused mass mortality events in both wild and cultured hard clams since the 1960s, yet progress in our understanding of QPX disease outbreaks has been limited in part by poor understanding of the biology and ecology of the causative agent – an opportunistic thraustochytrid that was first cultivated in the 1990's, but only recently formally described as *Mucochytrium quahogii*. Whilst several methods have been able to detect *M. quahogii* in seawater and sediment, its abundance was typically too low for reliable quantification by those methods. In this study, the authors describe the development of a nqPCR SYBR-based assay for sensitive detection and quantification of *M. quahogii* from marine environmental samples following the Minimum Information for Publication of Quantitative Real-Time PCR Experiments (MIQE) guidelines. They then applied the nqPCR assay to a large set of sediment and seawater samples, confirming that *M. quahogii* is prevalent in the environment, as expected for an opportunistic pathogen, and that the *M. quahogii*-specific nqPCR assay is sensitive enough to quantify *M. quahogii* at its natural environmental abundance. The new assay represents a valuable tool to better understand *M. quahogii* dynamics in the environment and possible relationships with QPX disease outbreaks and provides a model to guide the development of similar assays for other important marine microbes typically present at similarly low abundance.

Traditionally, diatoms have been assessed using microscopy, which in some cases is not reliable or reproducible, and more recently high-throughput sequencing using phylogenetic markers, such as various 18S rDNA regions, are not always reliable in taxonomy studies and diversity surveys because they cannot distinguish between some taxa. In the report by Dermastia et al. the chloroplast-encoded *rbcL* marker was used in a series of monthly samples from the Gulf of Trieste, northern Adriatic Sea, to assess diatom diversity, and it was found to reliably detect more taxa compared to 18S-V9 metabarcoding or microscopy. Whilst the authors showed a very thorough community analysis obtained by *rbcL* and its implications for studies dealing with taxa distribution and population structure, as well as carbon and silica flux models and networks, the authors highlight the incompleteness of reference databases and options for possible improvements in this regard.

The article by Neu and Kuhlicke describes a lectin-based approach to visually capture and analyze the hydrated structure of biofilms and bioaggregates, which has been a significant challenge. The authors present lectin data in form of a barcoding table that can serve as a guide to scientists for the selection of lectins in studies on glycoconjugates of multispecies and environmental biofilm/bioaggregate systems.

Finally, in the study by Mazière et al. a mesocosm approach is proposed on how to expose microbial mats, as a microbial community model, to changes in temperature and pH in order to investigate the impact of climate change on microbial communities representative of coastal areas. Although the method cannot represent the full spectrum of environmental complexity, it closely mimics *in situ* conditions and could be improved in this respect by, for example, adding rainfall cycles or seasonal variations.

## Author contributions

TG: Conceptualization, Funding acquisition, Validation, Writing – original draft, Writing – review & editing. FC: Conceptualization, Validation, Writing – review & editing. KZ: Conceptualization, Validation, Writing – review & editing.

